# Amino Acids, Peptides, and Proteins: Implications for Nanotechnological Applications in Biosensing and Drug/Gene Delivery

**DOI:** 10.3390/nano11113002

**Published:** 2021-11-08

**Authors:** Simge Er, Ushna Laraib, Rabia Arshad, Saman Sargazi, Abbas Rahdar, Sadanand Pandey, Vijay Kumar Thakur, Ana M. Díez-Pascual

**Affiliations:** 1 Biochemistry Department, Faculty of Science, Ege University, Bornova-Izmir 35100, Turkey; simgeer89@gmail.com; 2 Department of Pharmacy, College of Pharmacy, University of Sargodha, Sargodha 40100, Pakistan; ushnalaraib@yahoo.com; 3 Department of Pharmacy, Quaid-i-Azam University, Islamabad 45320, Pakistan; rabia.arshad@bs.qau.edu.pk; 4 Cellular and Molecular Research Center, Research Institute of Cellular and Molecular Sciences in Infectious Diseases, Zahedan University of Medical Sciences, Zahedan 9816743463, Iran; sgz.biomed@gmail.com; 5 Department of Physics, Faculty of Science, University of Zabol, Zabol 538-98615, Iran; 6 Department of Chemistry, College of Natural Science, Yeungnam University, 280 Daehak-Ro, Gyeongsan 38541, Korea; sadanand.au@gmail.com; 7 Biorefining and Advanced Materials Research Centre, Scotland’s Rural College (SRUC), Kings Buildings, Edinburgh EH9 3JG, UK; Vijay.Thakur@sruc.ac.uk; 8 School of Engineering, University of Petroleum & Energy Studies (UPES), Dehradun 248007, Uttarakhand, India; 9 Universidad de Alcalá, Facultad de Ciencias, Departamento de Química Analítica, Química Física e Ingeniería Química, Ctra. Madrid-Barcelona, Km. 33.6, 28805 Alcalá de Henares, Madrid, Spain

**Keywords:** amino acids, proteins, peptides, nanomaterials, drug delivery, gene delivery, detection

## Abstract

Over various scientific fields in biochemistry, amino acids have been highlighted in research works. Protein, peptide- and amino acid-based drug delivery systems have proficiently transformed nanotechnology via immense flexibility in their features for attaching various drug molecules and biodegradable polymers. In this regard, novel nanostructures including carbon nanotubes, electrospun carbon nanofibers, gold nanoislands, and metal-based nanoparticles have been introduced as nanosensors for accurate detection of these organic compounds. These nanostructures can bind the biological receptor to the sensor surface and increase the surface area of the working electrode, significantly enhancing the biosensor performance. Interestingly, protein-based nanocarriers have also emerged as useful drug and gene delivery platforms. This is important since, despite recent advancements, there are still biological barriers and other obstacles limiting gene and drug delivery efficacy. Currently available strategies for gene therapy are not cost-effective, and they do not deliver the genetic cargo effectively to target sites. With rapid advancements in nanotechnology, novel gene delivery systems are introduced as nonviral vectors such as protein, peptide, and amino acid-based nanostructures. These nano-based delivery platforms can be tailored into functional transformation using proteins and peptides ligands based nanocarriers, usually overexpressed in the specified diseases. The purpose of this review is to shed light on traditional and nanotechnology-based methods to detect amino acids, peptides, and proteins. Furthermore, new insights into the potential of amino protein-based nanoassemblies for targeted drug delivery or gene transfer are presented.

## 1. Introduction

Amino acids (AAs) have been spotlighted in research works over different scientific areas in chemistry and biology [1]. AAs and derived chemicals have gained substantial attention in drug development because of their fundamental roles in cells′ pathological and/or physiological processes [2]. As building blocks of various proteins, hydrophobic or hydrophilic AAs possess extraordinarily diverse features, including reverse cross-linking, chirality, and charge density [3].

AAs are traditionally classified as nutritionally essential or unessential for humans [4]. It has been reported that AAs were used as a supplementary therapy to treat many disorders [5,6]. Because of their low molecular weight and inefficient pharmacokinetics, they are not very effective in the clinic, which is a practical barrier that needs to be addressed [6]. Several artificial peptides and proteins consisting of essential AAs have been synthesized and successfully tested for biomedical applications [3]. Advances in nanotechnology have led to novel biosensing and therapeutic modalities for managing multiple diseases [7,8,9,10,11,12,13]. Nanomedicine is an emerging interdisciplinary field that involves nanotechnology, biology, and medicine. In this regard, the design of novel proteins that can be self-assembled into various supramolecular complexes is crucial in nanotechnology [14,15,16]. A variety of nanotherapeutic approaches have been recently introduced for biological applications, including to overcome chemotherapeutic resistance of cancer cells, combat cancer metastasis, etc. [11,13,17,18,19,20,21,22,23,24,25,26,27,28,29,30,31,32,33]. Furthermore, thermosensitive magnetic nanomaterials were exposed to alternate magnetic field in order to develop effective chemotherapeutic approaches [34,35,36,37,38].

Multiple biophysical methods can further characterize the stable form of these artificial nanoarchitectures [39]. Compared to free AAs, self-assembled nanostructure complexes composed of AAs have enhanced pharmacokinetic profiles and have shown increased accumulation in specific target sites [6]. Peptide and protein nanotechnology have demonstrated outstanding potential for the mimicry of living matter constituents and helped achieve novel materials by combining proteins/peptides with nonbiological components [40,41,42,43].

Researchers have recently exploited molecular imprinting procedures to design new polymer scaffolds that serve as synthetic receptors [44]. These can bind to specific organic chemicals, which proved valuable in developing biosensors [44,45]. In this context, arrays of nanostructures (i.e., carbon nanotubes, gold nano/microislands, etc.) with imprinted polymers have been prepared to detect AAs or proteins [44,45]. Moreover, metal-based nanoparticle (NP) sensors [46] and electrospun carbon nanofibers [47] have shown advantages in the electrochemical determination of AAs or peptides.

In addition, nanotechnology has introduced many innovative devices that serve as drug delivery and gene delivery systems [48,49]. In this regard, several AAs, peptides, and proteins have been studied for targeted drug delivery [50,51]. The attention to them partly stems from their flexibility in binding with different polymers and biological components [52]. Moreover, because of their low toxicity and facilitated cellular uptake, multifunctional protein-based nanocarriers hold great promise for the delivery of nucleic acids, such as DNA, short-interfering RNA (siRNA), etc. [53,54]. Through this review, we hoped to cast light on the nanotechnology-based techniques for sensing AAs/peptides/proteins and provide new insights into exploiting protein-based nanoassemblies for targeted delivery of specific drugs or genes.

## 2. Routine Methods for Detection of AAs, Proteins, and Peptides

Proteins are complex molecules essential to life that have enzymatic, structural, and storage functions. The most common techniques used to determine the total amount of protein are isotope ratio mass spectrometry (IRMS), the Kjeldahl method [55], and biuret methods such as the Lowry′s method [56] and the Bradford method [57]. Among them, the IRMS and Kjeldahl methods are susceptible and reproducible. However, artifacts have been observed in these methods. The interference effect is relatively high in spectrophotometric and colorimetric techniques used to determine the total protein amount. Therefore, the desired protein must be purified in the first step. However, this results in the loss of some proteins. None of the abovementioned methods provides information about AA composition.

The importance of AA analysis is increasing daily in different fields such as biochemistry, clinical chemistry, nutrition, and pharmaceutical formulation. The AA contents, chemical forms, and sample matrices (food, biological fluid, or protein hydrolysis) of many samples are quite different. AAs play a significant role in forming vital biomolecules such as hormones, neurotransmitters, antibodies, and signaling molecules. Since AAs are the precursors of many biomarkers, determining the amount of AAs in biological fluids is essential for the early diagnosis of many diseases. Studies have reported that many AAs play a role in forming diseases such as phenylketonuria, citrullinemia, and homocystinuria diseases [58,59].

Determining the separation and amount of AAs is very important to provide information about polypeptides’ and proteins′ characterization and structural properties. However, these compounds are difficult to identify and separate because of their high polarity and lack of strong chromophoric groups. Since many commonly used AAs cannot be determined directly by spectroscopic methods (UV–visible spectrophotometry or fluorometry), the amino groups of AAs are selectively modified with substances that show fluorescence or visible-light absorption prior to their determination [60]. Mass spectrometry (MS) and chromatography combination are currently used as analysis platforms. The separation and quantitative analysis of free AAs before or after protein hydrolysis is carried out with the aid of modern methods such as ion-exchange chromatography (IEC), gas chromatography/mass spectrometry (GC/MS), and liquid chromatography-mass spectrometry/mass spectrometry (LC–MS/MS). Each method comes with its own advantages and disadvantages.

Using the GC/MS method instead of GC with flame ionization or electron capture makes AA analysis more attractive. GC provides short analysis times, but AAs need to be derivatized into GC-detectable forms. However, this process also prolongs the analysis time. Substances such as N,O-bis-(trimethylsilyl), trifluoroacetamide (BSTFA), or N-methyl-N-(trimethylsilyl) trifluoroacetamide (MSTFA) can be used for derivatization. Still, steric hindrance due to the formation of bulky groups can be developed [61]. In 1998, Husek described rapid derivatization (about 1 min) of AAs with alkyl chloroformates. In this method, the esterification of carboxylic acids, amino groups, and hydroxyl groups was carried out to form alkyl esters or N(O)-alkoxycarbonyl ethers, and AA analysis could be performed in less than 10 min [62,63].

Moore and Stein were the first to develop an IEC-based AA analyzer in the 1960s [64]. In today’s methods, IEC and gas/liquid chromatography techniques are applied using different detectors. IEC coupled to the postcolumn ninhydrin derivatization method is the most widely used technique in the clinical field. It is considered a gold standard for detecting AAs in biological samples because of its wide dynamic range and linearity. The major disadvantage is that it is a time-consuming method (usually 2–3 h per sample) that requires high sample volumes (>200 µL). In addition, detecting interfering compounds that react with ninhydrin and cannot be determined by spectrophotometric detection generates problems [65,66]. The LC-MS/MS technique has become a compelling tool because of its better selectivity and shorter analysis times compared to IEC. In 2018, Casado and coworkers aimed to develop an ultraperformance liquid chromatography–tandem mass spectrometry (UPLC–MS/MS) procedure to identify 25 AAs and 17 related compounds in plasma, urine, cerebrospinal fluid (CSF), and dried bloodstains. The comparison of the results obtained from this procedure with those derived from IEC revealed a good correlation between the two techniques except for 4-hydroxyproline, aspartate, and citrulline [66]. In 2020, Carling and coworkers investigated and compared the analytical performance of three commercially available reagent kits for LC–MS, IEC, and LC–MS/MS, used for plasma AA analysis. According to their results, the LC–MS test showed a low correlation with IEC, while LC–MS/MS showed a good correlation with IEC. It was stated that IEC should no longer be defined as the gold standard method for plasma AA analysis, as LC-MS/MS offered superior specificity and faster analysis time. Although the sensitivity of the chromatographic techniques is high, they are expensive, do not allow point-of-care analysis, and require killed personnel. Detection of proteins by direct protein electrochemistry makes them suitable for ‘point of care’ or ‘in-field testing’ applications. Also, the electrochemistry of direct protein enables the detection of conformational changes and modifications in proteins [67].

## 3. Different Nanomaterials as Nanosensors for Detecting AAs, Proteins, and Peptides

Nanomaterials are promising materials with at least one size in the range of 1–100 nm. Outstandingly high surface areas can be attained via the intelligent design of nanomaterials. Furthermore, nanomaterials can be synthesized with outstanding electrical, optical, catalytic, and mechanical properties that are superior to those of their bulk counterparts. Nanomaterial properties can be adjusted as desired via controlling the synthesis conditions and adequate functionalization [68].

Nanomaterials can be categorized into three classes according to their content: (i) organic-carbon-based nanomaterials-carbon nanotubes (CNTs), carbon nanofibers (CNFs), fullerenes (C60), and graphene (GR). Chemical vapor deposition (CVD) [69], laser ablation [70,71], and arc discharge techniques [72,73] are used for the production of organic-carbon-based nanomaterials; (ii) inorganic-based nanomaterials—quantum dots, gold NPs, and magnetic NPs. These nanomaterials can be synthesized into metals such as Au or AgNPs, metal oxides such as TiO_2_ and ZnO NPs, and semiconductors such as silicon and ceramics; (iii) hybrid nanomaterials, which can be any combination of carbon-based, metal-based, or organic-based nanomaterials with any form of metal, ceramic, or polymer bulk materials [74].

**Figure 1:**
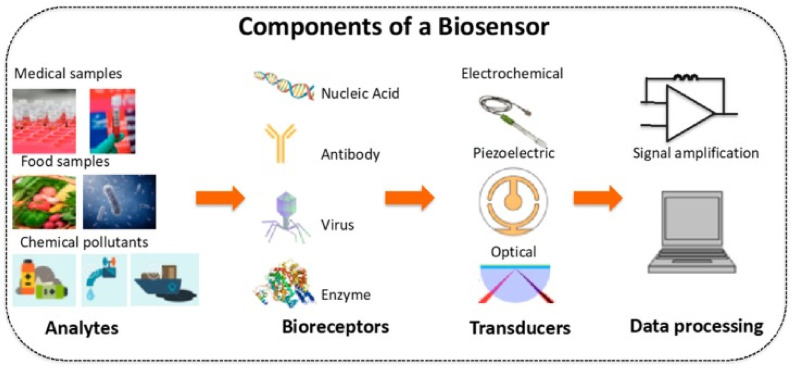
Schematic diagram of a typical biosensor. Reprinted with permission from ref. [76].

A sensor is an analytical device that can detect and quantify the presence of an analyte in a sample. It includes receptors, transducers, and reading systems. The biological receptor interacts specifically with the target analyte, and the transducer converts this information into a measurable signal [75]. For example, piezoelectric transducers are involved in measuring the change in mass after the formation of analyte–bioreceptor complexes, while optical transducers and electrochemical transducers measure the changes in light intensity and conductivity, current, or potential, respectively. Finally, the magnitude of the change is measured by the reading system. Figure 1 shows a schematic diagram of a typical biosensor.

Bio-based analysis systems have recently become the most used and desired devices for diagnosis in the clinical field because of their fast response times and reliable features. In biosensors, a biological element (the receptor) is immobilized on the transducer using different strategies [77]. Analyte detection is performed using the high affinity between the receptor and its ligands, such as antigen-antibody, enzymatic (enzyme–substrate), or cellular (microorganisms, proteins) interactions. The ability to detect important biomarkers such as nucleic acids, AAs, and proteins associated with a disease is essential for the clinical field [78]. The technique of immobilization of the biofunctional component on the working electrode dramatically affects the performance of biosensors. It is important to note that a biosensor’s stability is not lost while forming a close relationship between the biological component and the sensor surface (transducer). Therefore, the selection of immobilization matrices that support the performance of the sensor system is very critical.

Nanosensors are sensing devices with at least one sensing size smaller than 100 nm [79]. The use of nanoscale materials as reinforcement increases the interface area of the resulting composites. For this reason, various reinforcement elements such as hydroxyapatite, gold NPs, GR, CNTs, and CNFs are used to increase the surface area and especially the conductivity in sensor applications [80]. Carbon-based nanomaterials are widely used as reinforcements because of their stable, mechanically robust, flexible, electrical, and thermally conductive properties. Thus, these nanomaterials are promising in the development of high-performance devices [81].

Macro- and microscale sensors such as electrochemical and optical sensors are currently being used in the clinical field. For example, electrochemical and optical sensors such as blood gas and pH are frequently used in intensive care. Likewise, disposable electrodes are used in the clinical field to record biopotentials such as electrocardiograms and electroencephalograms [82]. Nevertheless, the use of nanosensors in the early-stage diagnosis of diseases and preclinical studies is increasing. In particular, whole-cell behaviors, adhesion processes of cells to the extracellular matrix, and cell-cell interactions can be easily monitored in vitro thanks to label-free electrochemical nanosensors [83]. For example, in vitro studies can be performed in the presence of components (drug or toxic substance) that can affect cell adhesions to the biofunctional surface of a nanosensor developed on a cell-based platform under the electrochemical measurements. This sheds light on the studies carried out before the transition to in vivo applications, which is the next step of preclinical studies. This also reduces animal experiments by using these developed nanosensors. At the same time, nanosensors are attracting much attention as an alternative to the invasive methods currently used to diagnose diseases in the clinical field. Recently developed wearable nanosensors are promising for noninvasive monitoring of biomarkers. It is crucial that some compounds that serve as disease biomarkers can be determined from saliva, sweat, or tears. At the same time, electrochemical nanosensors with increased stability are being developed for real-time monitoring of small molecules in blood or drug-active substances in plasma in a continuous flow environment [84].

### 3.1. Metal NP-Based Sensors

With the development of nanoscience and nanotechnology, metal NPs are highly desirable in areas such as nanosensors, biomedicine, biological labeling, and microelectronics because of their unique properties such as sizeable surface-to-volume ratio and high electrical conductivity, biocompatibility, catalytic activity, etc. [85]. Signal-generating molecules are usually used to bind bioreceptors to the biosensor recognition surface for labeling. Enzymes such as horseradish peroxidase are labeled agents and require an additional dye or substrate in affinity-based sensors. Enzyme labels are not stable, since they are affected by environmental conditions. Additionally, they are expensive. Nanoprobes have become quite popular as an alternative. Usage of electroactive NPs as nanolabels contributes to improving biosensor performance. Furthermore, electroactive NPs are inexpensive and stable [86].

Gold NPs (AuNPs) are widely used as colorimetric aptasensors, electrochemical aptasensors, and fluorescent aptasensors because of their high extinction coefficient and chemical stability, strong localized surface plasmon resonance absorption, and optical properties. Since AuNPs show different colors according to their size and morphology, they are used to detect analytes such as proteins and small molecules by using colorimetric techniques. The combination of AuNPs with specific ligands is quite common [87,88]. In 2017, Khezri and coworkers developed a nanosensor by using the inner filter effect (IFE) of AuNPs on CdS quantum dots (QDs) to detect arginine. This AA caused an increase in the size of the NPs due to their aggregation. Changing color (red to blue) triggered turn-on of the IFE-decreased CdS QDs’ fluorescence. The linear detection range of the CdS QD/AuNP system for detecting arginine in human serum and other samples containing arginine was 7–215 μg L^−1^, and the limit of detection (LOD) was found to be 2.4 μg L^−1^ [89]. In 2018, Hai and coworkers developed a nanosensor using AuNPs in core-shell structures combined with reduced graphene quantum dots (r-GQDs) to detect cysteine. The resulting core-shell AuNPs@r-GQDs exhibited an intensive surface plasma band at 525 nm due to their excellent dispersion. Cysteine was used as the crosslinking agent that triggered the aggregation of AuNPs@r-GQDs, leading to a color change. Based on this, the colorimetric determination of cysteine was performed, and the LOD in human plasma was found to be 5.6 nM [90]. Bai and coworkers developed an ultrasensitive electrochemical sensor to determine Mycobacterium tuberculosis IS6110 fragment (MTB) based on AuNPs with modified C60 NPs/nitrogen-doped graphene nanosheets as a signal enhancer [91]. In this study, nitrogen-doped graphene nanosheets modified with nano-C60 and AuNPs showed high conductivity and improved redox activity. The developed electrochemical biosensor showed a broad linear detection range for MTB detection (10 fM–10 nM). The LOD of the developed DNA biosensor system was determined as 3 fM. In 2020, Beitollahi and coworkers developed a label-free aptasensor using AuNPs for the detection of homocysteine. In this study, homocysteine-binding-aptamer (HBA) was immobilized on an AuNP-modified glassy carbon electrode (Au/GCE) surface to produce an aptasensor. The linear detection range of the system was found to be 0.05–20.0 μM, while the LOD was determined as 0.01 μM [92]. In 2021, Morawski and coworkers created an electrochemical platform to assess norepinephrine and dopamine in human blood serum and urine samples using mesoporous silica/titania (SiTi) and AuNPs. It was reported that surface modification of SiTi material with AuNPs led to a significant improvement in low charge transfer resistance and redox peak current. The LODs were 0.35 μmol L^−1^ and 0.57 μmol L^−1^ for norepinephrine and dopamine, respectively [93].

Although silver NPs (AgNPs) offer better properties than AuPs, they are less desirable in sensor applications because of their lower chemical stability. However, recent studies have been carried out to strengthen the chemical stability of AgNPs. The advantages of these nanoparticles include their low cost and an efficient combination with proteins by reacting with their thiol group (–SH) [94]. Zhu and Lee developed a sandwich-type immunosensor for the detection of α−1 antitrypsin (AAT), a biomarker of Alzheimer’s disease. The developed biosensor was based on 3,4,9,10-perylene tetracarboxylic acid/carbon nanotubes (PTCA–CNTs) as a sensing surface, and AgNPs modified with alkaline phosphatase-labeled AAT antibody (ALP-AAT Ab–Ag NPs) as a signal tag. The peak current values obtained by using AgNPs in this sandwich-type immunosensor system were much higher than the peak current values obtained in the absence of the NPs. These results were proof that the AgNPs improved the sensor performance as signal enhancers. The linear detection range for AAT was 0.05–20.0 pM, and the LOD was 0.01 pM [95]. Kumar and Sundramoorthy developed an AgNP-decorated nitrogen-doped single-walled carbon nanotube-modified glassy carbon electrode (GCE) for nonenzymatic electrochemical detection of urea, a non-protein nitrogen compound. The linear detection range of the developed sensor system was 66 nM–20.6 mM, and the LOD was 4.7 nM [96]. In 2019, Meng and coworkers prepared a peptide cleavage-based electrochemical biosensor to detect prostate-specific antigens using graphene oxide and AgNPs for signal generation. Nyquist diagrams proved that AgNPs effectively supported the electron transfer rate. PSA concentration was determined from the electrochemical signal change that occurred because of the cleavage of the specific peptide used on the sensor surface in the presence and the absence of PSA. The linear detection range of the developed sensor system was 5 pg mL^−1^–20 ng mL^−1^, and the LOD was 0.33 pg mL^−1^ [97]. One year later, Awan and colleagues designed a sandwich-type immunosensor by antibody functionalized-silver-NPs (Ab–AgNPs) to determine NS1 (dengue biomarker). The linear detection range using AgNPs as signal enhancers was 3–300 ng mL^−1^, and the LOD for NS1 detection was 0.5 ngmL^−1^ [86]. In 2021, Nycz and coworkers prepared an electrochemical biosensor based on AgNPs and titanium urea dioxide nanotubes to determine heat shock protein 70 (HSP70) as a potential tumor marker. Titanium dioxide (TiO_2_) exhibits outstanding properties such as biocompatibility, large surface area, high stability, and good electrical conductivity [98]. Usage of TiO_2_ nanotubes with AgNPs increased the electrical conductivity of the sensor system, thereby improving its analytical performance. The linear detection range of the developed biosensor was 0.1–100 ng mL^−1^, and the LOD was 0.48 ng mL^−1^ [99].

NPs such as platinum (PtNPs) cause a compatible effect with hydrogen peroxide (H_2_O_2_) on electrocatalytic activity to increase electrical conductivity, catalytic activity, and biocompatibility. Thus, rapid diffusion of target analytes occurs on the electrochemical biosensor surface, where the enzyme or antibody is immobilized. A sandwich-type electrochemical immunosensor was developed by Liu and coworkers for alpha-fetoprotein (AFP) detection using PtNPs anchored on cobalt oxide/graphene nanosheets (PtNPs/Co_3_O_4_/graphene). The combination of these nanomaterials resulted in better electrochemical performance and improved catalytic activity for reducing H_2_O_2_. The linear detection range of the developed electrochemical immunosensor was 0.1 pg mL^−1^–60 ng mL^−1^, and the LOD was 0.029 pg mL^−1^ [100]. The following year, Gao et al. synthesized a novel label-free electrochemical immunosensor for the detection of monocyte chemoattractant protein-1 (MCP-1) by using single-walled carbon nanohorns (SWCNHs) functionalized with PtNPs (PtNPs–SWCNHs). After modification of SWCNH with PtNPs, antibody immobilization efficiency and electron transfer rate effectively increased due to the increased surface area and conductivity of PtNPs. Furthermore, high catalytic activity for the reduction of H_2_O_2_ was obtained in the presence of these NPs. The linear detection range of the developed electrochemical immunosensor was 0.06–450 pg mL^−1^, and the LOD was 0.02 pg mL^−1^ [101]. Similarly, Thirumalraj and coworkers developed an electrochemical sensor based on PtNPs supported graphite/gelatin hydrogel to determine H_2_O_2_ in biological samples; the sensor showed improved electrocatalytic activity and high sensitivity for the detection of this analyte. The linear detection range was 0.05–870.6 μM, and the LOD was 37 nM [102]. In 2020, Oliveira and colleagues developed a flexible platinum electrochemical immunosensor to detect Parkinson’s disease biomarkers (dopamine and the Parkinson’s disease protein 7 (PARK7/DJ-1). Pt is a noble metal that exhibits similar properties to Au; hence, Pt electrodes are a good alternative to Au electrodes. Pt electrodes are also cheaper compared to gold ones. In a study performed with Pt electrodes, the conductivity capacity results revealed that they had identical properties to those of Au electrodes. The linear detection range of dopamine detected by voltametric measurements was 3.5 × 10^−5^–8.0 × 10^−4^ mol L^−1^, and the LOD was 5.1 × 10^−6^ mol L^−1^. The linear detection range of PARK7/DJ-1 by electrochemical impedance spectroscopy was 40–150 ngmL^−1^, and the LOD was 7.5 ngmL^−1^ [103]. In 2021, Tian and coworkers developed a dual-aptamer biosensor for detecting COVID-19 nucleocapsid protein (2019-nCoV-NP) by using metal-organic frameworks MIL-53(Al) modified with enzymes and Au@PtNPs. Firstly, the Au glassy electrode (GE) surface was modified with two thiol-modified aptamers (N48 and N61). Subsequently, the nanomaterial-based composites (Au@Pt/MIL-53(Al)) were synthesized, and HRP and hemin/G-quadruplex DNAzyme were used as modification agents. This nanoprobe was developed to amplify the signal of the aptasensor by the increased hydroquinone oxidation in the presence of H_2_O_2_. Finally, the nanoprobe with protein-aptamer was developed on the GE surface. The linear detection range of the developed sandwich-type electrochemical sensor system was 0.025–50 ng mL^−1^, and the LOD was 8.33 pg mL^−1^ for early diagnosis of 2019-nCoV-NP [104].

### 3.2. Carbon-Based Nanomaterials

Carbon-based nanomaterials display outstanding properties such as high electrical conductivity, fast electron transfer capability, and high specific surface area, making them highly interesting for developing high-performance biosensors [105]. Commonly used carbon nanomaterials are carbon nanotubes (CNTs) and graphene and its derivatives, in the forms of nanotubes and platelets, respectively.

#### 3.2.1. Carbon Nanotubes

**Figure 2:**
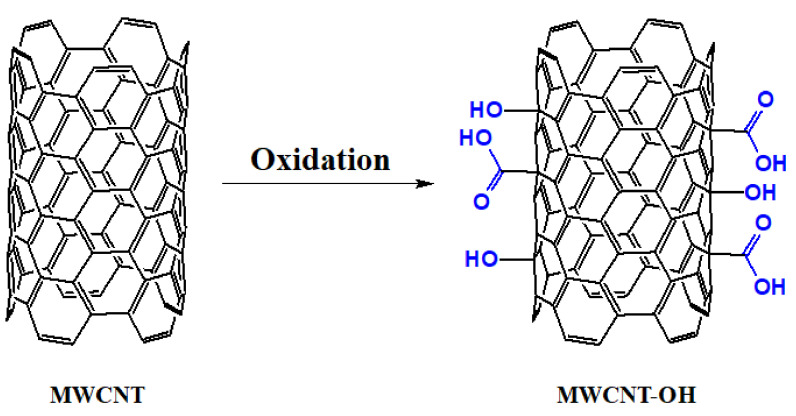
The oxidation process of MWCNT.

CNTs increase the sensitivity of biosensor systems by allowing a large number of biomolecular interactions on their large surface area while reducing the response time through their excellent electrical conductivity. Delocalized π electrons in the Z-axis give CNTs unique electrical properties. Therefore, they are potential candidates for label-free point-of-care protein detection [106]. Single-walled carbon nanotubes (SWCNT) are obtained by rolling a single layer of graphene, and multiwalled carbon nanotubes (MWCNT) are prepared by rolling multiple layers of graphene. The van der Waals forces between the CNT layers and the presence of highly polarized π-electron clouds cause CNT aggregation. The oxidation of CNTs leads to the formation of carboxyl and hydroxyl groups on their surface. Thus, their hydrophilicity, level of exfoliation, and solubility increase in polar media. Furthermore, different chemical agents can be used to further modify their surface via linking/interaction with these groups [107]. Figure 2 demonstrates the oxidation process of MWCNT.

**Figure 3:**
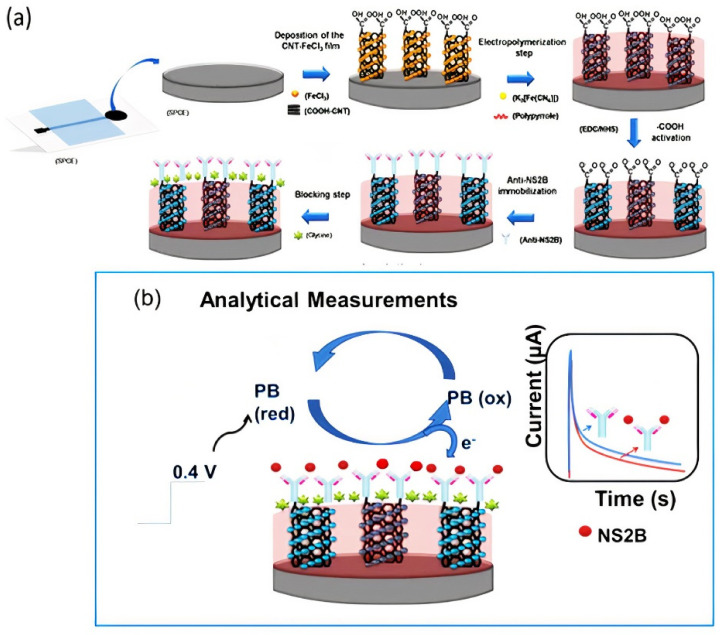
Schematic representation of ZIKV immunosensor. (**a**) Electrode preparation stage and (**b**) principle of analytical measurement. Reprinted with permission from ref. [112].

In 2017, Reddy and coworkers developed an electrochemical sensor based on functionalized multiwall carbon nanotube–chitosan biopolymer nanocomposite (Chit–fCNT) to detect epinephrine (Epn) in urine and pharmaceutical samples. MWCNTs were oxidized using nitric acid to yield fCNT with hydroxyl groups. Then, a bionanocomposite was prepared by dispersing the fCNTs in a chitosan solution, which was deposited on the electrode surface to conduct electrochemical measurements. The linear detection range of the developed electrochemical sensor system was 0.05–10 μM, and the LOD was 30 nM for Epn detection [108]. In 2018, Sobhan et al. developed a biosensor based on SWCNTs by using linear sweep voltammetry (LSV) measurements to detect the protein Ara h1, which induces peanut allergy. In this regard, 1-pyrenebutanoic acid succinimidyl ester (1-PBSE) was used to link the SWCNTs and Ara h1 antibody through noncovalent bonding (π-π stacking interactions). The linear detection range of the developed biosensor was 1–1.000 ng mL^−1^, and the LOD was 1 ng mL^−1^ for protein Ara h1 detection [109]. The following year, Dudina and coworkers developed a monolithic biosensor platform by using carbon-nanotube field-effect transistors (CNTFETs) for glutamate determination. The CNTFETs were functionalized with glutamate oxidase through 1-ethyl-3-(3-dimethylaminopropyl) carbodiimide (EDC) and N-hydroxysulfo-succinimide (sulfo-NHS). The developed biosensor showed a detection range between 250 and 500 µM, and the LOD was 10 µM [110]. In 2020, Palomar and coworkers prepared an electrochemical sensor based on peptide-modified AuNP/CNTs to detect a proteolytic enzyme named matrix metalloproteinase-7 (MMP-7). This enzyme is overexpressed in cancer and cardiovascular diseases. Enzymatic cleavage of peptides immobilized on the nanocomposite-modified electrode surface was carried out to increase the system’s stability. The linear detection range of the developed biosensor was 1 × 10^−2^−1 × 10^3^ ng mL^−1^, and the LOD was 6 pg mL^−1^ [111]. Recently, Silva and coworkers developed a label- and probe-free immunosensor based on Prussian blue@carbon nanotube–polypyrrole (PB@CNT–PPy) nanocomposite for the determination of the NS2B protein of Zika virus (ZIKV) by using an amperometric technique. Modification of a carbon nanotube–polypyrrole composite with Prussian blue (PB) caused redox catalytic activity. Thus, electrochemical analysis was performed without adding any redox probe solution to the medium (probe-free detection). Covalent immobilization anti-NS2B was carried out through EDC/NHS chemistry on the screen-printed electrode (SPCE). This sensor had the advantage of using a point-of-care diagnosis. Good selectivity was obtained between negative and positive ZIKV serum using this immunosensor (Figure 3) [85].

#### 3.2.2. Graphene-Based Nanomaterials

Graphene (GR) is a two-dimensional (2D) carbon nanomaterial with a single atomic layer of sp^2^ carbon atoms arranged in a honeycomb lattice. Owing to its high surface area and high electrical conductivity, it has a high potential to be the world’s thinnest electrode material used in electrochemical applications. In addition, GR, with its extensive π-electron system, has a strong affinity for carbon-based cyclic structures found in drugs, pollutants, and biomolecules. GR has a specific surface area of ~2630 m^2^ g^−1^, larger than that of CNTs (1315 m^2^ g^−1^) and raw graphite (10 m^2^ g^−1^) [113]. Furthermore, the electrical conductivity of GR is 60 times higher than that of SWCNTs [114]. Since GR-modified electrodes exhibit a wide electrochemical potential window, the determination of molecules in a wide potential range is feasible.

**Figure 4:**
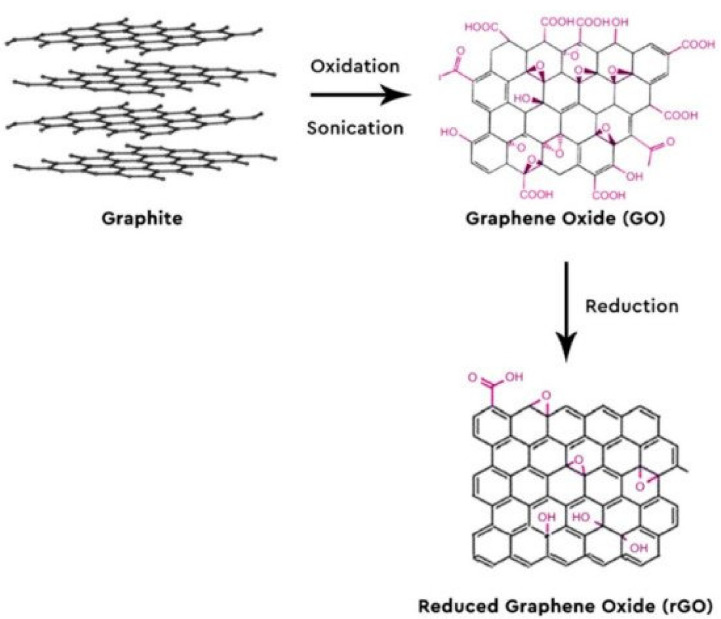
Oxidation and reduction steps to obtain reduced graphene oxide (rGO) from graphite. Reprinted with permission from ref. [117].

For this reason, GR is the most commonly used material for electrodes in biosensors [115]. GR has two main derivatives with different degrees of oxidation: graphene oxide (GO) and reduced graphene oxide (rGO). The increased solubility of GO in an aqueous solution is mainly due to its functional groups such as epoxides, hydroxyls, and carboxylic acids. These surface functional groups can interact with the functional groups of other biomolecules to be attached to their surface, providing many reaction sites [116]. However, the presence of oxygen-rich functional groups in GO causes a decrease in electrical conductivity. In this regard, GO is reduced with the desired modification for sensor applications. After reduction, most of the functional groups in GO are removed, and π-conjugation-rich graphene is formed, which is called rGO. Thus, the conductivity of graphene is restored via π-conjugation, although its solubility in aqueous solutions or polar solvents decreases. Figure 4 depicts the oxidation and reduction steps to synthesize rGO from graphite.

In 2017, Settu and coworkers developed an aptamer biosensor to detect engrailed-2 (EN2, a biomarker for prostate cancer) based on carboxylated SPCE. The reactive surface area was increased with the incorporation of GR into a carbon paste electrode. This increased the electrical conductivity, resulting in the development of a high-sensitivity biosensor system. The linear detection range was 35–185 nM, and the LOD was 38.5 nM. However, the calculated LOD value was not enough for the clinical diagnosis of EN2 protein. Therefore, more research on signal enhancement is needed to improve the LOD [118]. One year later, Baluta and coworkers prepared an electrochemical biosensor to sense epinephrine (EP) by using graphene quantum dots (GQDs) and glassy carbon electrodes (GC) modified with laccase. Catecholamine was oxidized in the presence of the laccase, and an electrochemical signal was obtained. The linear detection range was 1 × 10^−6^–120 × 10^−6^ M, and the LOD was 83 nM, to detect EP in labeled pharmacological samples [119]. The following year, Karimi and coworkers developed a molecularly imprinted polyaniline-based sensor with rGO to determine human cardiac troponin T (cTnT). MIP was obtained via electropolymerization on the rGO-modified SPCE in the presence of cTnT and carboxylated aniline monomers. Then, cTnT was removed by oxalic acid solution. The linear detection range of the developed biosensor was 0.02–0.09 ng mL^−1^, and the LOD was 0.008 ng mL^−1^. This study suggests that the developed biosensor system and HPLC have an excellent correlation [120]. In 2020, Sharma and coworkers (2020) developed a label-free aptasensor based on rGO modified with polyethylenimine (PEI) thin films for detection of cardiac myoglobin (cMbi, cardiac biomarker). PEI, a cationic polymer, was used for the reduction of graphene oxide (GO). In this way, a positive charge was formed on the rGO surface. The negatively charged single-stranded DNA aptamers were directly immobilized to the sensor surface by electrostatic interaction without any binding agent. The linear detection range of the developed biosensor was 0.001−1000 ng mL^−1^, and the LOD was 0.97 pg mL^−1^ (phosphate-buffered saline) and 2.1 pg mL^−1^ (10-fold-diluted human serum) for detection of cMb [121]. More recently, Jozghorbani and coworkers produced a label-free immunosensor based on rGO to detect carcinoembryonic antigen (it is well known that labeling methods may lead to steric hindrance on the electrode surface). The linear detection range of this biosensor was 0.1–5 ng mL^−1^, and the LOD was 0.05 ng mL^−1^, to detect carcinoembryonic antigen. In addition, the developed sensor was examined in human blood serum for CEA detection, and the results correlated well with those obtained using the standard enzyme-linked immunosorbent assay (ELISA) [122].

### 3.3. Electrospun Nanofibers (ESNFs)

**Figure 5:**
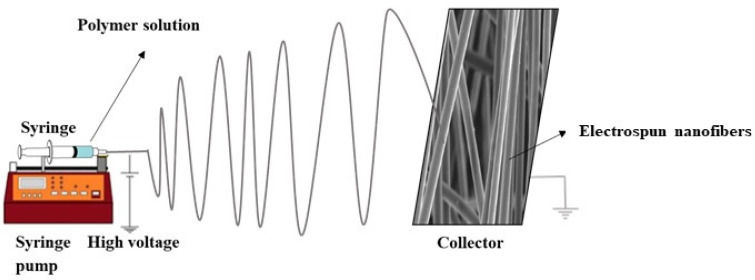
Representation of a conventional electrospinning setup.

Electrospinning is defined as the production of nanofibers from polymer solutions under a high electric field (kV) [123]. It is the only method for mass production of continuous long nanofibers [124]. Among the numerous nanomaterials, ESNFs are building materials in drug delivery systems, biosensors, biomedicine, food textile, and environmental applications because of their large surface areas, controllable surface conformations, porous structures, and high concentrations adsorption capacity, and good biocompatibility [125,126,127]. Because of these properties, electrospun nanofibers have better sensitivity than sensors formed with other materials. In addition, biomimetic coatings can prevent biofouling, thereby extending the life of biosensors [128]. ESNFs are produced via electrospinning, which is a simple, effective, controlled, and economical method. Fibers can be obtained from various materials; solutions or melt forms of organic polymers are among the most common sources. In particular, the production of nanofibers is possible from composite materials obtained by the appropriate combination of components with different morphologies in the nano size (e.g., NPs, nanorods, nanowires, nanotubes, and nanosheets) with organic polymers. Figure 5 shows a schematic representation of a conventional electrospinning setup.

In 2017, Soares and coworkers developed two different immunosensors by using electrospun polyamide 6 and poly(allylamine hydrochloride) nanofibers assembled with CNTs and AuNPs for the determination of the biomarker CA19-9. The detection limits calculated using impedance spectroscopy were 1.84 and 1.57 U mL^−1^ for electrospun nanofibers containing MWCNTs and AuNPs, respectively [129]. In the following year, Wang and coworkers developed an electrochemiluminescence (ECL) immunosensor to determine p53 (TSP53, tumor suppressor protein). AuNP-decorated, MWCNT-doped chitosan (CTS) electrospun nanofibers (MWCNT–CTS) were used for antibody (CAb) immobilization for the detection of TSP53. The linear detection range of the developed ECL immunosensor was 1 pg mL^−1^–1 ng mL^−1^, and the LOD was 0.5 pg mL^−1^ to detect the carcinoembryonic antigen in normal human cubital vein blood samples [130]. Two years later, Asmatulu and coworkers developed label-free electrochemical nanobiosensors to determine cyclooxygenase-2 (COX-2) in human serum samples and phosphate-buffered saline (PBS) using polyaniline nanofibers. Fibers with different average diameters (256, 481, 575, and 641 nm) were fabricated using the electrospinning technique to compare their nanobiosensor performance, which was examined using electrochemical impedance spectroscopy (EIS). The MWCNT–CTS–AuNP nanofibers were used as a supporting immobilization matrix for antibody (CAb) to detect TSP53 in PBS and human serum solutions. The LODs were 0.01, 0.1, 1.0, and 50.0 pg mL^−1^ for the nanofibers with the diameters of 256, 481, 575, and 641 nm, respectively. The highest sensitivity was obtained for the lowest average diameter of 256 nm because of its increased surface area [131]. In 2020, Arshad and coworkers developed a molecularly imprinted polymer (MIP)-based impedimetric sensor to detect NS1 (nonstructural protein 1, a specific biomarker for dengue virus infection). Polysulfone (PS) nanofibers were used for the modification of SPCE. Dopamine was used as a monomer, and self-polymerization was carried out in the presence of NS1 (template molecule). The linear detection range of the developed biosensor was 1–200 ng mL^−1^, and the LOD was 0.3 ng mL^−1^, for sensing NS1 in real human serum samples [132]. In 2021, Gobalu and coworkers developed a nanobiosensor system using biotin–aptamer linker immobilization on molybdenum disulphide/cellulose acetate (MoS2/CA) nanofiber composite for the detection of troponin I by EIS. Troponin I was detected up to 10 fM with a stability value of 90% after 6 weeks [133].

### 3.4. Molecularly Imprinted Polymers

Molecular imprinting is a promising method for developing affinity-based nanomaterials with high specific recognition ability [134,135]. Molecularly imprinted polymers (MIPs) provide many properties such as selectivity, stability, reusability, and low cost compared with biological recognition materials such as enzymes and antibodies. They have some drawbacks, such as a high diffusion barrier and low space accessibility, given that most of the imprinted areas are formed inside the MIP. To overcome these issues, the surface printing technique, which involves the production of a MIP layer on the surface of nanomaterials, has been developed in recent years. This method provides the advantages of higher bonding capacity and faster bonding kinetics on the material surface [136]. The applications of MIPs combined with electrochemical studies have increased in the sensor field because of their ease of use and low cost [137]. However, some problems still need to be overcome before MIP-based sensors can enter the sensor market. The most significant change is in the distance of the imprinted cavities to the sensor surface and, accordingly, low signal reception [138]. Therefore, researchers have focused on improving the surface of nanosized support materials such as GR with ultrathin polymeric films. Through this method, higher selectivity is provided for thin MIP layers [115]. In 2017, Cheng-Jun and coworkers developed a MIP-based electrochemical sensor using the C-terminal polypeptide of insulin as a template molecule and o-phenylenediamine (o-PD) as a functional monomer via electropolymerization on an Au electrode for the determination of insulin. The steric hindrance on the electrode surface was reduced by using C-insulin polypeptide as a template molecule instead of insulin. The linear detection range of the developed biosensor was 1.0 × 10^−14^–5.0 × 10^−13^ M, and the LOD was 7.24 × 10^‒15^ M for the detection of insulin. Furthermore, good selectivity and stability were obtained with the developed sensor in serum samples [139]. The next year, Parlak and coworkers developed another MIP-based wearable organic patch-type electrochemical device for noninvasive real-time cortisol determination from sweat. A cortisol imprinted biomimetic polymeric membrane was coated on top of poly(ethylenedioxythiophene):poly(styrenesulfonate) (PEDOT:PSS) by spin coating. The performance of molecularly selective organic electrochemical transistors (MS-OECTs) was examined, and the results were compared with those of non-molecularly selective organic electrochemical transistors (NS-OECTs). Rapid response time of less than 1 s was obtained by using the MS-OECTs, while the NS-OECTs did not give any response to increased cortisol concentration. Furthermore, MS-OECTs exhibited reversibility of the binding process. For measurements performed with both ex situ and wearable MS-OECTs, the developed wearable sensor system showed a sensitivity of 2.68 µA dec^−1^ (current per order of magnitude in cortisol) over the range of 0.01 to 10.0 µM cortisol concentrations. Simultaneously, the MIP-based wearable sensor developed in the selectivity studies carried out in the presence of analogs in sweat, which may interfere with the system, showed a good selectivity [140]. In 2019, Sun et al. used MIPs and a hybridization chain reaction to develop microfluidic paper-based analytical devices (μPADs) to detect glycoprotein ovalbumin (OVA). First, a SiO_2_@Au/dsDNA/CeO_2_ composite was used as a signal tag. The use of SiO_2_@Au improved the electron transfer efficiency and provided a larger surface area. Hybridization chain reaction (HCR) was carried out in the presence of two hairpin DNAs to obtain double-stranded DNA (dsDNA) on the SiO_2_@Au surface. Boronate affinity-based MIPs were prepared on the μPAD surface in the presence of Au nanorods (NRs) and 4-mercaptophenylboronic acid. 1-naphthol was used as a redox-active catalytic amplifier for the electrochemical measurement. The linear detection range was 1 pg mL^−1^–1000 ng mL^−1^, and the LOD was 0.87 pg mL^−1^, for the detection of OVA [141]. In 2020, Mugo and coworkers produced another MIP-based flexible electrochemical sensor for detecting cortisol in sweat. Cortisol-imprinted poly(glycidylmethacrylate-co ethylene glycol dimethacrylate) (poly(GMA-co-EGDMA)) was synthesized. The sensor layers consisted of stretchable polydimethylsiloxane (PDMS) based on carbon nanotube–cellulose nanocrystal (CNC/CNT) conductive nanofilms. The cortisol-imprinted poly(GMA-co-EGDMA) was synthesized as a cortisol biomimetic receptor on the CNC/CNT. The linear detection range was 10–66 ng mL^−1^, and the LOD was 2.0 ± 0.4 ng mL^−1^. The MIP sensor also exhibited high specificity in the presence of glucose, epinephrine, β-estradiol, and medroxyprogesterone as selected interfering species [142]. Raziq and colleagues prepared a portable electrochemical sensor based on a MIP film with ncovNP to sense SARS-CoV-2 antigen (ncovNP). The developed sensor was examined with samples of the nasopharynx swabs of patients. For this purpose, m-phenylenediamine was used as a monomer to obtain ncovNP-imprinted polymer, and 4-aminothiophenol (4-ATP) was used as a modification agent for thin-film electrodes with gold (Au-TFE). 3′-dithiobis (sulfosuccinimidyl propionate) (DTSSP) was used as the cleavable linker monolayer on the 4-ATP/Au-TFE surface to yield the ncovNP–MIP film. The linear detection range was 0–111 fM, the LOD was 15 fM, and the limit of quantitation (LOQ) was 50 fM [143].

## 4. Peptide, Protein, and AA-Based Nanomaterials for Targeted Drug Delivery

**Figure 6:**
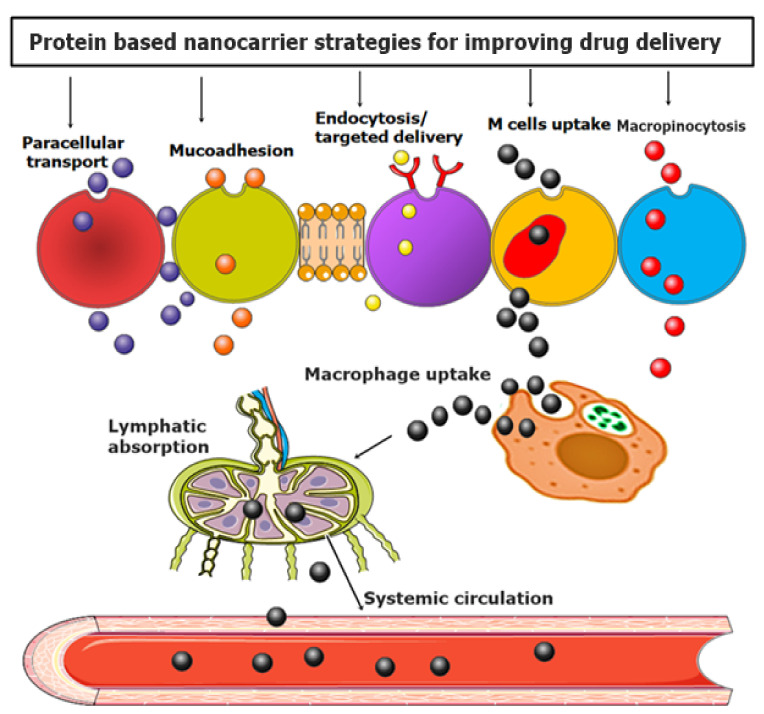
Visual representation of various strategies employed in using protein-based NPs for enhancement of targeted drug delivery.

Proteins, amines, and peptide-based drug delivery are opening new eras for drug delivery according to the synergism of nanotechnology [53]. It has been proved that protein-based ligands are excellent targeted agents with multifaceted features of biodegradability, stability, biocompatibility, and most importantly, flexibility in binding with various biological agents and polymers to develop multifunctionalization [52,144]. The most essential features of AAs, peptides, and proteins for targeted drug delivery are given in Table 1. Moreover, a visual representation of various strategies employed in using NPs to enhance intracellular drug delivery across the mucosal membrane is depicted in Figure 6.

### 4.1. Glutathione Nanocarriers

Glutathione (GSH) is a tripeptide that helps induce strong antioxidant action via reversing the damages caused by reactive oxygen species (ROS) [145]. The utilization of GSH as a ligand in drug delivery is highly productive for multidimensional diseases [146,147]. Therefore, polyethylene glycol and polypropylene sulfide block copolymer (PEG–PPS) were synthesized by Wu et al. [148] through previously developed methods. PEG–PPS block copolymer was further attached to the S-nitroso-glutathione (GSNO) prodrug, and its release was triggered by ROS and GSH. The concept of this strategy can be utilized for reversing the chemoresistance in tumors via increasing targeted accumulation of the drug in the tumor via following the mechanistic approaches of ROS and GSH. The amphiphilic conjugate of the PEG–PPS–GSNO was attached to the doxorubicin (DOX) therapeutic moiety. The DOX-loaded amphiphilic nanocarriers were successfully synthesized and characterized for NP size estimation, and amphiphilic polymer conjugation was confirmed by NMR, FTIR, and gel permeation chromatography. In vitro dissolution, cell cytotoxicity, biocompatibility and chemosensitivity of DOX were also evaluated. However, most importantly, the cellular uptake studies were carried out via various advanced techniques like confocal microscopy, flow cytometry, and in-vitro. Flow cytometry for analyzing apoptotic cell death was also performed. Overall, it was proved that GSNO nanocarriers showed the highest loading capacity for NO, stabilized, and redox-triggered drug release in the tumor microenvironment with improved biocompatibility. These multifunctionalized GSH tripeptide-based NPs can serve as effective codelivery platforms for NO and DOX in the targeted killing of chemoresistant cancer cells by inducing chemosensitivity [148].

### 4.2. Transferrin-Linked Polymeric Nanocarriers

Leukemia is a blood cancer categorized by genetic mutations in the development of leucocytes, which heavily damaged the bone marrow and lymphatics by triggering the hematopoietic stem cells in uncontrolled proliferation of bones, thus producing immature leucocytes [149,150]. Among other anticancer drugs, DOX is still preferable in treating leukemia, but its therapeutic potential is compromised by its induced nonspecified cardiotoxicity and poor solubilization. Therefore, Fang et al. [151] developed a novel protein-based ligand and transferrin functionalized biocompatible polymeric nanocarrier system for advanced treatment against leukemia. First, a novel polymeric block was synthesized composed of distearoyl phosphatidylethanolamine (DSPE) and polyethylene glycol (PEG). The DPSE–PEG block polymer was conjugated with transferrin (TF) protein to achieve tumor-targeted delivery. The transferrin ligand was immobilized onto the polymeric block conjugate to finally form DPSE–PEG–TF, and DOX was subsequently added to yield DPSE–PEG–TF–DOX. The transferrin-conjugated nanocarriers were characterized via physical analysis, dissolution, cell viability, NPs uptake, and TF targeting assays. Furthermore, activated partial thromboplastin time (APTT) and prothrombin time (PT) assays were performed in parallel with hemolysis and apoptosis assays.

The results showed that TF-functionalized nanocarriers had a spherical morphology with a hydrodynamic size of 80 nm for 75% drug encapsulation. Moreover, the essential feature was the DOX release in the intravacuolar compartments following endocytosis, which improved targeting efficiency [151].

### 4.3. Polydopamine-Layered Zein Nanocarriers

Glioblastoma multiforme (GBM) is a damaging primary tumor of the brain, causing several morbidity and mortality cases worldwide [152]. GBM is often resistant to conventional therapies. However, a significant barrier in successful drug delivery is the blood-brain barrier (BBB) that bypasses the chemotherapeutics’ intratumoral delivery [153]. Zein is currently important because of its safety, biodegradation capabilities, and sustained drug release characteristics [154]. Novel research indicated that surface functionalization of zein NPs with polydopamine (PD) layers resulted in enhanced solubility, biocompatibility, stability, and flexibility for attachment of various biological functional groups. However, curcumin has been proven to induce strong anticancer activity. Therefore, Zhang et al. [155] developed polyamine-rich protein zein-based nanocarriers for efficient, targeted therapy against GBM. In this research, curcumin was attached with PD-layered zein NP to form (CUR–Z–PD) NPs through a modified phase separation technique. Furthermore, CUR–Z–PD NPs were characterized for size determination and other physicochemical features, transcytosis assay, uptake mechanistic features in deep glioma cells, ROS determination, apoptosis, cell migration assay, different antimicrobial assays, and intravesicular quantification of zein functionalized NPs in zebrafish larvae. After a detailed set of experimentation, it was concluded that the NPs markedly inhibited the proliferation and migration in glioma cells and increased cellular uptake and ROS production with induced apoptosis in the glioma cells, approaching efficient therapy against GBM.

### 4.4. Poly-L-Lysine Based Lipid Self-Emulsifying Nanocarriers

**Figure 7:**
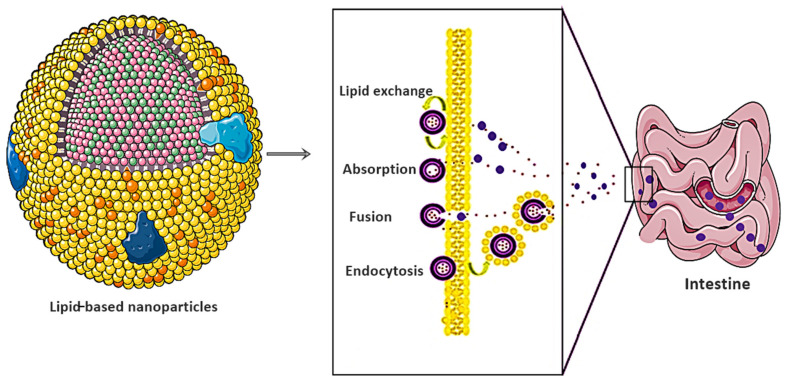
Mechanism followed by lipid-based nanocarriers for overcoming the intestinal barrier in order to improve targeted delivery against *S. typhi*.

*Salmonella typhi* (*S. typhi*) resistant strains are a significant economic and public health burden for developing and underdeveloped countries [156]. Moreover, all classes of antibacterial drugs showed resistance owing to nontargeted delivery and poor solubilization. Arshad et al. introduced the unique concept of indulging cell-penetrating peptide poly-l-lysine as a multifunctional flexible ligand for targeted M-cell therapy [157]. The authors further utilized lipid NPs as a vehicle for targeted drug delivery [158]. Lipid-based nanocarriers use the mechanistic approaches of lipid exchange, absorption, fusion, and endocytosis to overcome intestinal barriers, as shown in Figure 7. However, among other lipids, NPs, self-emulsifying drug delivery systems (SEDDS) have optimistic prospects owing to their easy industrial scaling and improved thermodynamic stability.

**Figure 8:**
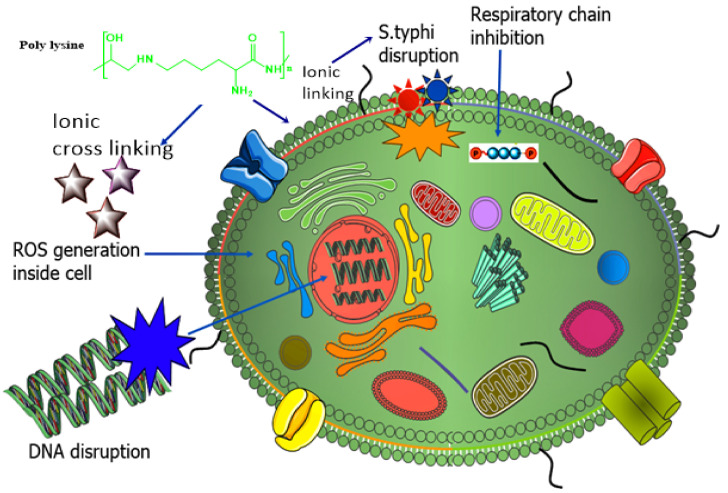
Mechanism of interaction of poly-L-lysine with the *Salmonella typhi*.

The strategy behind the synthesis of novel poly-L-lysine (PLL) SEDDS was to enhance highly specified targeted drug delivery against *S. typhi* by generating ROS and disrupting bacterial DNA [159,160], as shown in Figure 8. The researchers further conjugated PLL with mannose, preactivated hyaluronic acid, and Pluronic to develop amphiphilic conjugate PLL–M–PTHA–F127 via reductive amination. Biconjugation of mannose with PLL and hyaluronic acid resulted in advancement in treatment against *S. typhi*. Characterization tests, including physicochemical, in vitro, and in vivo tests, were performed. It was proved that enhanced recognition by receptor scavenging cells and intracellular trafficking facilitated the internalization of PLL multifunctionalized SEDDS of ciprofloxacin into intestinal epithelial cells, resulting in proficient targeting with the eradication of *S. typhi* and 100% survival. Moreover, the exciting fact relating to PLL is its capability of forming a stabilizing ligand for successful and targeted delivery of SEDDS in the intestine and increasing the efficacy of an antimicrobial drug via preventing multibacterial drug resistance [161,162].

### 4.5. Vancomycin-Loaded Thiolated Nanocarriers

Vancomycin belongs to a class of glycopeptide antibiotics produced by the actinomycete bacterium *Streptomyces Orientalis* that has bactericidal action for all Gram-positive bacteria, including methicillin-resistant staphylococcal strains (MRSA) [163]. According to reports, it is the most preferred drug for treating bacteria-related infections of *Staphylococcus aureus*, especially MRSA and other methicillin-resistant *Staphylococcus* strains [164]. Blepharitis is the anterior or posterior inflammation of eyelids, which can be subacute or chronic, caused by *S. aureus* and seborrheic bacteria [165]. Linezolid and vancomycin were reported to be most effective against the staphylococcus bacteria and overcame resistance towards penicillin, erythromycin, and ciprofloxacin [166]. The ocular barriers, such as involuntary eye muscle movement, tears, etc., remove foreign particles, including drugs, coming across the eye surface, which means that ocular drugs require frequent administration. To exert local effect to the cul de sac, overcoming these barriers is important, which can be addressed by increasing the time of retention of a drug in tears [167]. Ocular delivery systems have proven advantageous and preferred routes for local and systemic drug administration. Jahan et al. [168] addressed the above-related problems in the ocular delivery of drugs by fabricating thiolated Pluronic-based polymeric nanomicelles of vancomycin against blepharitis. Thiolated Pluronic-based vancomycin nanomicelles were successfully synthesized via thin-film hydration technique and characterized via physicochemical, in vitro, and in vivo histopathological assays. The initial results of this research indicated that these vancomycin nanomicelles were effective targeted ocular delivery systems against staphylococcal blepharitis with improved retention time, sustained drug release, and targeted anti-inflammatory action.

### 4.6. Arginine-Based Nanocarriers

Multidrug resistance is the primary cause of the severity of infectious diseases such as *Salmonella typhi.* Pathogenic organisms have developed various resistance mechanisms such as genetic mutations, target site modifications, enzyme inactivation, and efflux pump activation [149]. In 2017, Mudakavi et al. developed arginine-coated nanocarriers by conjugating L-arginine (Arg) with pectin and protamine, followed by complete coating with mesoporous silica NPs (MSNs) through a layer-by-layer coating method. Arginine is also crucial for targeting infectious diseases such as *Mycobacterium tuberculosis* and *S. typhi* because of its innate cellular responses against macrophages. However, it is also a dietary component of the *S. typhi* pathogen. In cellular responses, arginine produces nitric oxide (NO), inducing cytotoxic activities in macrophages against *S. typhi*. As far as the conceptualization of the uptake regulation of arginine is concerned, *S. typhi* infections lead to the upregulation of cationic transporters, which is accountable for augmented uptake of arginine. Therefore, the Arg–MSN-based nanocarriers of ciprofloxacin were successfully synthesized and characterized for size, shape, zeta potential, localization of NPs, cellular trafficking, and uptake via advanced confocal microscopy and in vivo survival assays [169]. However, detailed experimentation decreased bacterial burden and increased survival because of synchronized antibacterial, targeted, and ROS cellular response against *S. typhi*.

## 5. Peptide, Protein, and AA-Based Nanomaterials for Targeted Gene Delivery

Gene delivery systems are essential for treating gene disorders in humans via gene therapy [170]. Gene therapy can be explained as transferring genetic material directly to tissues and cells to treat acquired or inherited disorders [171]. The optimal results of a gene delivery system depend on the customization and targeting of the respective system. Usually, a gene is inserted into affected patients in lieu of drugs or surgeries. Other approaches that have been utilized include:

- Replacement of a mutated gene with a healthy gene;
- Introduction of new genes;
- Knocking out malfunctioning mutated genes.

A gene delivery system usually has three parts or constituents:

- A gene encoding a particular therapeutic protein;
- A plasmid-based gene expression system, which regulates the behavior of genes within the targeting cell;
- A system for controlled delivery of gene expression plasmid to the targeted site in the body [170].

### 5.1. Proteins as Nanomaterials for Gene Delivery

The use of proteins and peptides for synthesizing and assembling functional nanomaterials is an active area of research. A large variety of nanoscale materials with interesting properties can be developed by merging molecular biology and biochemistry. These bioenabled materials offer more advantages over their nonbiological counterparts [172]. Protein-based nanocarriers are of particularly great interest because of their renewable sources. They provide reduced cytotoxicity, while the uptake to target cells is significant. Hence, these protein-based nanomaterials are promising candidates for gene delivery [53]. Nucleic acids such as siRNA, mRNA, or pDNA have promising applications therapeutically. Therapies based on nucleic acids are versatile because of their design, which offers promising treatments. Nevertheless, a particular delivery system is required for their delivery [54].

AAs are building blocks of structures such as proteins and peptides. Around 20 naturally occurring AAs exist; they enable the synthesis of these structures in living cells. AAs are sequenced together via amide linkages or peptide bonds, leading to thousands of proteins that differ in structure and functions. A one-letter code usually refers to the primary structure provided by the AA sequence. These AAs can be classified into hydrophilic, hydrophobic, charged, and other categories, depending on the characteristics of the ‘R’ group. Hence, a relationship exists between the AA sequence and the structure. This accounts for the fact that the specific configuration depends on the R groups that are close to each other in a peptide chain. The endless number of sequences can be explained because even a short peptide of 5 AAs has about 3.2 million possible sequences. However, despite this fact, a minority of peptide sequences are utilized in biological systems [173,174].

Proteins can be obtained from plants and animals. Only those relevant to gene delivery are discussed herein. Among various examples of proteins, gelatin is commonly utilized in gene delivery [175]. It is a denatured protein that can be obtained by alkali or acid hydrolysis of collagen, has been safely used in pharmaceuticals in the past, and has been regarded as GRAS (generally recognized as safe) by the Food and Drug Administration (FDA). Furthermore, it is a polyampholyte, since it contains both anionic and cationic groups. The helical structure of gelatin is due to the repeating sequence of glycine, proline, and alanine AA triplets [53]. Gelatin NPs have been used successfully in gene therapy in the past [176,177].

A group of researchers designed poly-siRNA-thiolated gelatin (psi-tGel). First, they prepared tGel NPs, then polymerized the siRNA with thiol groups. The polymerization occurred at 5′ of both sense and antisense strands, resulting in enhanced interactions between siRNA and tGel. The researchers demonstrated the efficacy of psi(RFP)-tGel NPs for gene silencing induction in RFP/B16F10 melanoma cells [178]. In another study, Moran et al. used gelatin B and protamine sulfate (PS) to deliver DNA. Gelatin B is fascinating, since it becomes negatively charged at physiological pH because of its isoelectric point in the range of 4.8–5.2. This results in interactions with molecules of opposite charges. When gelatin B comes in contact with an endosome, its charge becomes positive, thus releasing therapeutic agents. For efficient gene delivery, protamine sulfate traps the DNA inside the gelatin B–PS complex. This is attributed to the highly positive charged PS, which binds DNA. The researchers also showed that two things affected the release of DNA: (i) the gelatin’s gel strength and (ii) the initial concentration of DNA [179].

Albumin is utilized for assisting other molecules in gene delivery. The primary sources of albumin are human serum albumin (HSA) or bovine serum albumin (BVA) [180]. It is the main protein of blood plasma and has various reactive groups on its surface that aid in easy modifications. Its ability to accumulate in tumors makes it an innovative cellular carrier [170]. Prajapati et al. wrote a detailed review on different kinds of albumin nanocarriers and highlighted different approaches for enhancement of transfection efficiency as well as targeted delivery to tumor sites by modification of albumin surface [181]. Karimi and coresearchers used a core-shell structure to design a novel Alb–CS–DNA complex. The core and shell were made of albumin and chitosan, respectively, and show interactions with DNA. The fabricated complex was introduced into HeLa cells to deliver plasmid shRNA (short-hairpin RNA) against the GL3 luciferase. Their results indicated that the synthesized complex NPs were present in 85% of HeLa cells with minimal toxicity. They also suggested that albumin imparted biocompatibility to the complex NP compared to plain Alb–NP or CS–NP [182]. Han et al. synthesized cationic bovine serum albumin (CBSA) by modifying the surface of BSA with ethylenediamine. Mixing siRNA with CBSA caused electrostatic interactions that led to the formation of CBSA/siRNA NPs. The results demonstrated efficient delivery of siRNA to B16 lung metastatic cells. Also, CBSA protected siRNA from RNA degradation [183].

Elastin is a protein that provides elasticity and exists in connective tissues. For applications in gene delivery, both the ELPs and α-elastin have been used. The artificial peptide, ELP, has a protein sequence (Val-Pro-Gly-*X*-Gly)*_n_*, where ‘*X*’ can be any AA and ‘*n*’ is the number of repeating units [180]. Dash et al. synthesized a dual ELP-based injectable system for the delivery of two different genetic cargos. The dual system consisted of an ELP gel scaffold and ELP hollow spheres, previously used in gene delivery. The dual system contained two different plasmids for modulation of angiogenesis and inflammation to treat critical limb ischemia. One plasmid, contained in the ELP gel scaffold, encoded interleukin-10 (IL-10), while the other plasmid, in the ELP hollow spheres, encoded eNOS (endothelial nitric oxide synthase). The results indicated that release occurred in a controlled manner with reduced inflammation and increased density in a blood vessel [184,185].

Silk is another protein obtained from the silkworm *Bombyx mori* and spiders (*Nephila clavipes* and *araneus diadematus*). The AAs present in silk are highly repetitive, which causes mechanical characteristics in silk [186]. Current investigations have demonstrated ultrathin silk fibroin (SF) as a potential gene delivery system [187]. For example, Li et al. designed SF vector using a polystyrene template. The purpose of designing SF was transfection of NIH/3T3 fibroblasts via pDNA. Optimal coatings of SF required for adsorption of pDNA were determined by zeta potential. The researchers suggested the efficiency of plasmid DNA loaded onto SF microcapsules for transfecting fibroblasts. They also indicated that the transfection efficiency was affected by the method of loading DNA, either pre- or post-SF deposition [188].

Zein is a plant protein found in maize seeds. Plant storage proteins (prolamine) contain high levels of proline, an AA. As a result of its high AA content, zein has hydrophobic side chains, making it insoluble in water. The sustained delivery of DNA has been achieved using this property [189]. Zein is also considered a GRAS polymer by the FDA. Researchers extended the work of Regier et al. [190], synthesized zein nanofibers, and showed controlled release of siRNA for up to 72 h in skin fibroblasts for gene silencing. Gene silencing was reported after 72 h due to the presence of a significant amount of siRNA entrapped in fabricated nanofibers [191].

### 5.2. Peptide-Based Nanomaterials for Targeted Gene Delivery

Designing nanomaterials with peptides is already well established as a versatile method. Two approaches that had been successful. First, one involved the exploitation of AAs with specific properties as chemical moieties. Second, based on the concept of natural motifs, AA sequences can be utilized to design or create structures [174].

The past years have seen a rapid increase in the synthesis and development of peptide-based nanomaterials. The applications of these nanostructures are trending in gene therapy because of their properties such as biological barrier penetration, high stability, enhanced loading rate, and targeting ability. For genetic therapy, AA monomer-based peptides with amide bonds are considered principal units in the development of bionanomaterials. Because of different ‘R’ groups, different AA has different structures and functions [192].

### 5.3. Combination of Peptide-Based Nanomaterials with Different Molecules for Genetic Delivery

**Figure 9:**
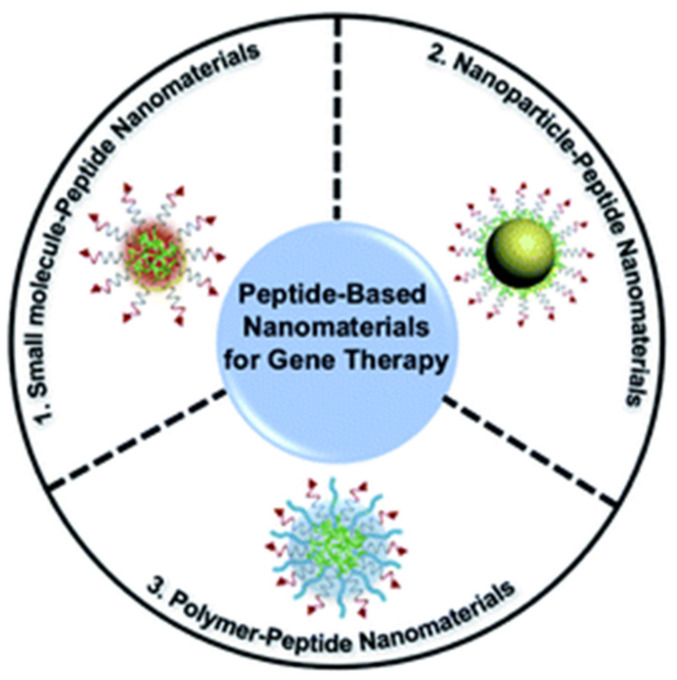
Peptide-based nanomaterials for genetic delivery. Reprinted with permission from ref. [192], Copyright 2021, Royal Society of Chemistry.

Three individual functional components and peptide-based self-assembled nanomaterials have been discussed for gene delivery (Figure 9). Unlike pure peptides, these peptide-based nanomaterials show good biocompatibility, high loading rate, and good multifunctionality [192].

#### 5.3.1. Small Molecules

Small molecules can reach the target area because their diffusion through cell membranes is easy. The efficiency of the transfection, targeting ability, and loading capacity of peptide-based nanomaterials can be improved by the ability of small molecules and peptides that are positively charged to combine with nucleic acids that are negatively charged [193,194,195]. AIEgens (aggregation-induced emission iluminogens) are small fluorescent molecules that emit fluorescence at a high aggregated state and do not require concentration control [196]. A group of researchers found out that covalently bonded peptides and AIEgens exhibited properties of both substances, i.e., the biological properties of peptides and the luminescence of AIEgens. They fabricated self-assembled NPs, called TNCP/ASO-NPs, by peptide-conjugated AIEgen (TNCP) and ASO to deliver antisense single-stranded DNA oligonucleotide (ASO) efficiently. The AIEgen part of TNCP was hydrophobic PyTPE, which promoted the self-assembly of NPs between 76 and 198 nm. The peptide sequence can be further divided into three parts:

i) DGR or RGD, for targeting integrin αvβ3;
ii) KRRRR, a nuclear localization sequence, siding the entry of antisense oligonucleotide into the nucleus;
iii) RRRR, a cell-penetrating peptide, for aiding in endosomal escape and assisting NPs to enter cells.

The correlation coefficient of ASO-Cy5 and TNCP, along with microscopy techniques and in vivo testing, demonstrated successful delivery of ASO to a tumor target site in mice and inhibition of Bcl-2 expression for tumor growth inhibition [197]. The same group carried out further research and showed that a triple combination therapy, named FCsiRNA-PyTPA, efficiently stopped tumor growth by down-regulating the expression of antiapoptotic proteins [198].

Kostorelos et al. prepared self-assembled peptide nanofibers (PNFs) using palmitoyl and peptide (GGGAAAKRK) and reported the ability of the prepared self-assembled PNFs to silence Bcl-2 in loci of the brain by delivering siRNA [199].

#### 5.3.2. NPs

Certain NPs, such as gold, porous silicon, and nanodiamonds, are used in gene therapy for their specific good characteristics. Combining NPs with peptides can further enhance the efficacy while reducing toxicity [200,201]. Strouse et al. designed a solid AuNP complex for genetic delivery into MSC (mesenchymal stem cells) of rats utilizing Ku70 peptide. Ku70 peptide is a pentapeptide from Ku70, a DNA repair binding sequence. The researchers modified the surface of AuNPs with BDNF/mCherry fusion gene (6.6kbp) and the N-terminal of Ku70 peptide with cysteine via thiol linkage. This instigated the development of an AuNP complex with a size of about 130 nm. Modification of the Ku70 peptide made it zwitterionic, which aided in reducing electrostatic interactions between the fusion gene and the AuNP peptide surface. This enhances the efficacy of the gene’s transfection. Different analyses and in vivo testing showed that the AuNP complex inhibited apoptotic response [202].

Another group of researchers fabricated cationic functionalized nanodiamonds to increase cellular uptake and deliver antisense oligodeoxynucleotides (ODNs) to the nucleus. Cationic TAT–NLS peptides were used to modify 30 nm nanodiamonds (NDs). Then, ANA4625 nucleic acid was loaded via electrostatic interactions. Optical imaging, MTT assay, and Western blot analysis were performed. The results indicated ANA4625 loaded in TAT–NLS–NDs inhibited Bcl-xL and Bcl-2 gene expression through enhanced cytotoxicity in MCF-7 cells. Hence, the designed TAT–NLS–NDs proved to be more efficient carriers than uncoated NDs [203]. Lang et al. used cell-penetrating peptides (CPPs) with magnetic NPs (MNPs, FE3O4) for transfection of plasmid (pGL3), SCO, and siRNA [204].

#### 5.3.3. Polymers

Specific polymers are easily precipitated, and aggregation occurs in vivo because of their hydrophobic nature. Combining them with other polymers that are hydrophilic in nature forms self-assemblies that demonstrate in vivo stability and improved uptake by target cells due to enhanced hydrophilicity [192]. Leong and coworkers used cationic α-helical peptide (PPABLG) to self-assemble PEGylated NPs (P-HNPs). The purpose of designing it was to deliver Cas9 expression plasmid with sgRNA [205]. Unlike CPPs, PPABLG was able to bind and concentrate plasmid DNA and short siRNA enhanced endosomal escape and cellular internalization by maintaining the ability of increased membrane penetration. PEG-polythymine40 (PEG-T40) was used to modify sgRNA complexes and PPABLG-Cas9 expression plasmid to enhance stability extracellularly. Three pathways were found related to the internalization of P-HNPs: (i) caveolae-mediated uptake, (ii) micropinocytosis, and (iii) clathrin-mediated pathway. In vitro testing showed 46.2% cell apoptosis at the target site in HeLa cells by P-HNPPCas9+sgPlk1, while in vivo results and Western blot analysis confirmed tumor suppression greater than 71% and reduced the expression level of Plk1 protein up to 67% [205].

A great deal of research has been conducted on polymers because of their excellent properties. To deliver siRNA/microRNA for selectively targeting osteoblasts, Wang et al. used osteoblast targeting peptides to modify and develop polyurethane nanomicelles. The design was intended to avoid over toxicity and/or immune response [206].

Micelles, vesicles, nanotubes, NPs, and nanofibers can be combined with amphiphilic and peptide conjugates to yield nanoassemblies that offer a significant number of specific properties. Peptide-based nanomaterials are suitable candidates in biomedical applications such as carriers for gene delivery, as they combine the properties of both nanoscale systems and peptides and can conjugate or condense DNA/RNA [207].

Three primary constituents are present in these supramolecular structures:

i) hydrophobic AAs;
ii) hydrophilic AAs;
iii) positively charged AAs [208].

The hydrophobic AAs control the self-assembly and development of secondary structures via molecular interactions that are noncovalent in nature. The hydrophilic AAs residues impart stability while positively charged AAs electrostatically interact with the negatively charged nucleic acids [208]. However, if these interactions exist between negatively charged nucleic acids and positively charged peptide residues (lysine, histidine, and arginine), peptiplexes are formed spontaneously [209].

#### 5.3.4. Micelles

Peptide-based micelles can be described as nanoassemblies that are closed monolayers. The outer shell is hydrophilic, while the inner core is hydrophobic. Two general methods of their fabrication include solvent switch and direct dissolution. These well-ordered structures are spontaneously formed in nano range above CMC (critical micelle concentration) and are regulated by temperature [210,211].

These micelles offer significant properties in gene delivery systems, including high stability and a size range that facilitates deep penetration into tumors and cellular uptake. Their practical gene loading ability offers high therapeutic potency. It is important to note here that micelle nanoassemblies that are cationic in nature support efficient DNA condensation by increasing the positive charge density in the solution. Also, these cationic-based micelles can be customized to specific target cells and can facilitate endosomal escape, transport to the nucleus, and cellular uptake [212].

Ryu et al. synthesized self-assembling multifunctional peptides (MP, CR8GPLGVH5-Pal). Dimerization was performed to create a gene delivery system. The MPP can be self-assembled to prepare micelle structures, and pDNA condensation occurs via electrostatic interactions. The potential of MPP for use as a gene delivery platform was demonstrated by high transfection efficiency in cancer with high expression of MMP-2 [213].

Peptide-based micelles are now being designed as smart nanomaterials for tuning gene delivery. The stimuli responsiveness in these micelles should control the release of a gene, improve cellular uptake, and control the destiny of nucleic acids intracellularly [214,215,216]. For example, (Fmoc) 2KH7-TAT is a pH-responsive chimeric peptide that can mediate transfection of PGL-3 reporter plasmid with or without the existence of serum in 293T and HeLa cell lines. These pH-responsive micelles can synergistically deliver drugs and genes [217].

#### 5.3.5. Vesicles

Vesicles can be described as spherical assemblies that are bilayer delimited and hollow. Hydrophilic regions are exposed to external and interior aqueous environments, while the hydrophobic residues are packed together between hydrophilic interfaces [218]. Hydrophobic molecules are trapped between hydrophobic bilayers, whereas hydrophilic moieties are entrapped in the inner aqueous phase [219]. Adjustment of chain length of building blocks and composition can tune the size of vesicles [220].

The assembly of peptides either into vesicles or nanotubes is governed by the hydrophobic nature of peptides’ tails. Surfactant-like peptides with hydrophobic tails consisting of 4–10 glycine residues and hydrophilic heads of aspartic acid were shown to self-assemble into vesicles. The diameter of the self-assembled vesicles was in the range of 30–50 nm. Peptide-based nanovesicles provide several advantages. However, targeting mediated by peptides and preservation of contents from extracellular factors is the prime factor for in vivo delivery of DNA. Organ distribution is improved if DNA stability is maintained and circulation time is prolonged [221,222].

Cationic SPVs (GE11-GHDC/HQCMC/Chol) were synthesized for the delivery of genes or siRNAs. These SPVs showed high zeta potential. Functionalization of GE11-GHDC-based vesicles demonstrated desirable properties, e.g., gene transfer, targeting of epidermal growth factor receptor (EGFR), and in vivo suppression of tumor growth with high potency [223].

Like micelles, peptide building blocks can be used to create smart vesicles responsive to external and internal stimuli. For example, poly (L-lysine hydrochloride) (PLL) and poly(gamma-benzyl-d7-L-glutamate) copolypeptides, upon combining with plasmid DNA, assembled to form stimuli-responsive vesicles, i.e., pH- and temperature-responsive nanocarriers. The increased protection of pDNA can be attributed to partial condensation on the PLL phase and partial encapsulation inside the formed vesicles [224].

#### 5.3.6. Nanofibers

Nanomedicine is the medical application of nanotechnology, ranging from the medical applications of nanomaterials and biological devices to nanoelectronic biosensors and even possible future applications of molecular nanotechnology such as biological machines [225,226,227]. Nanofibers (NFs) are long 1D cylindrical nanostructures usually 5-20 nm wide. They show a high loading capacity for nucleic acids owing to their high surface-to-volume ratio [208,228].

Peptides that can self-assemble into NFs include amyloid peptides, collagen-like triple-helical peptides, amphiphilic peptides, and ionic self-complementary peptides [229]. Interactions of the side chains and the secondary structure and the customization of AAs while contemplating hydrophilic–hydrophobic interactions play a significant role in the self-assembly and formation of NFs [230].

The aspects that confer distinct characteristics for gene delivery in peptide-based NFs (PNFs) are:

i) A hydrophilic head constituted of some positively charged essential AAs in physiological states;
ii) The capability of a peptide sequence that is responsible for β-sheet formation for intermolecular hydrogen bonding;
iii) A hydrophobic tail, primarily an alkyl chain [231].

Electrostatic interactions between negatively charged nucleic acids and positively charged AAs make PNFs a propitious tool for gene delivery. A group of scientists synthesized PNFs for siRNA delivery as a nonviral vector system. In vitro results showed effective destruction of Bcl-2 expression and generated apoptosis. In vivo administration of PNF/siRNA complexes to rat brain demonstrated enhanced biological activity and residence time of siRNA [199].

#### 5.3.7. Nanotubes

Peptide nanotubes (PNTs) are highly organized 3D systems. The amphiphilic building blocks maintain a cylindrical hollow shape via interactions at the molecular level [232]. PNTs are relatively new in nanomedicine research; therefore, few examples have been reported [233].

Ghadiri et al. first reported cyclic polypeptide-based organic nanotubes. Also, the transmembrane channels proposed PNTs as potential gene delivery systems into biological cells [234]. Another group of researchers synthesized an oral gene delivery system by self-assembly of nanotubes using cyclic cyclo-(D-Trp-Tyr) in the presence of pDNA. Results indicated increased duodenal permeability of pDNA in vitro and in vivo. The researchers also suggested the potential applications of these systems for genetic treatment of stomach, kidney, liver, and duodenum-related diseases [235].

Surfactants such as peptides can also self-assemble into these nanotubes. The hydrophilic tail is sequestered from contact with water through the generation of a polar interface, facilitating nanotubes’ assembly [236]. Researchers assembled nanotubes using surfactant-like peptides with hydrophobic tail (6 Ala, Val, Leu) residues and cationic heads (1-2 Lys and His) when the isoelectric point of a peptide was lower than the value of the pH. The synthesized PNTs were potential gene delivery systems because of their cationic nature, which binds negatively charged DNA or siRNA [237].

#### 5.3.8. Peptiplexes

Peptiplexes are formed via electrostatic interactions between positively charged peptide residues and nucleic acid’s negatively charged phosphate backbone. These complexes are compact and stable in nature and have been recognized as efficient carriers in the past years [238,239]. Compared to polyplexes or lipoplexes, peptiplexes offer many advantageous properties such as ease of synthesis at large scales, biocompatibility, stability in case of oxidation, and numerous customization possibilities [240,241]. As for the synthesis of peptiplexes, around six to eight positive charges per peptide are needed to condense pDNA into NPs. However, to form more stable peptiplexes, 13 or more positive charges are required [242]. Different combinations of AAs, such as histidine, arginine, and lysine in specific cationic peptides, have already been studied for condensing nucleic acids. Out of these examples, lysine-rich peptides are more efficient and strongly dependent on genetic cargo concentration. This was attributed to the existence of protonatable amine groups on these residues [243]. For example, nanosized peptiplexes were synthesized when branched amphiphilic peptides with oligo lysine segments condensed pDNA-encoded green fluorescent protein (GFP). The formation of peptiplexes occurred through strong electrostatic interactions at low peptide/pDNA ratios [244].

Arginine-rich peptides are also effective delivery systems because of compact gene condensation [245]. For example, siRNA and pDNA peptiplexes were formed using RALA. RALA has seven arginines in the backbone and is an amphipathic CPP [246,247,248].

Similarly, in the case of histidine residues, protonation of the imidazole ring occurs at low pH. As a result, endosomal escape and gene release occur, making it an efficient gene delivery mediator system. This DNA transfection efficiency can be increased by using branched peptides with higher histidine density than short linear peptides [242,249]. Interestingly, a combination of histidine and arginine improved transfection efficacy by promoting cell penetration of NPs [250].

K12H6V8, a cationic amphiphilic peptide used in genetic delivery, consists of three molecules:

i) A histidine block responsible for the endolysosomal release;
ii) A hydrophilic valine block;
iii) A DNA-binding lysine block [251].

### 5.4. Barriers in Using AAs, Peptides, and Proteins for Gene Delivery

It is important to consider certain aspects when delivering genes to humans, e.g., which carriers are required to transfer DNA into the target cell’s nuclei, whether the carriers are efficient enough for transfection, whether these can be safely used in humans, whether they can protect DNA from factors like degradation before it enters the target cell, and most importantly, whether they can deliver a gene to target cells and tissues.

The possible rate-limiting steps for efficient delivery of genetic cargo are intracellular and extracellular barriers. Nucleolytic degradation in the cytosol, lysosomal degradation, and inefficiency of delivering to nuclei are critical intracellular barriers [252]. Nucleolytic degradation in serum by the reticuloendothelial system (RES), along with nonspecific delivery, are included among extracellular barriers [253]. Gene vectors should be able to navigate through many intracellular and extracellular barriers to achieve high gene-transfection efficiency [254].

## 6. Summary and Outlook

The current review summarizes the latest advancements over the last five years in developing nanosensors to determine proteins, AAs, and metabolic biomarkers, including NPs, carbon nanotubes, graphene, electrospun fibers, and molecularly imprinted polymers. With the development of nanotechnology, the integration of nanosized materials into sensor systems has enabled the production of sensitive, low-cost analytical devices that do not require expert personnel and allow point-of-care analysis. Modifying a sensor surface with stable nanomaterials greatly improves the performance indexes of the system, such as sensitivity, stability, repeatability, and signal-to-noise ratio. The development of nanosensors offers significant advantages in the clinical field, especially as an alternative to systems with high-sensitivity gold standards such as GC–MS, LC-MS/MS, IEC, which are fairly expensive and do not allow point-of-care analysis.

Drug delivery has been radically improved by the application of proteins, AAs, and peptides. A new polymer with increased biocompatibility and tumor targeting abilities may help overcome numerous shortcomings of conventional delivery systems. Emerging trends of protein-based multifunctionalized nanocarriers with biocompatible and biodegradable polymers against various cancers and infectious diseases have tremendously improved drug delivery.

Nonviral vectors have attracted considerable interest because of their safety and stability profile as compared to viral vectors. Proteins, peptides, and AAs are not only renewable resources but abundant in nature. Nanomaterials based on these natural resources for targeted delivery of genetic load represent an active area of research. Several research and review articles in this regard have provided critical and valuable information. This review summarizes some protein-based nanomaterials for targeted gene delivery. It also highlights individual functional components of peptide-based nanomaterials and sheds light on different peptide-based nanoassemblies for genetic delivery. Although in vivo studies have shown promising results, more research is required to analyze the complex nature of AA sequences in proteins and peptides. Studies on the limitations of these nanomaterial-based genetic delivery systems are also necessary to advance clinical trials and approval by the FDA.

## Figures and Tables

**Table 1:** Critical features of various AA- and protein-based nanocarriers.

Nanocarrier	Key Feature	Ref.
Glutathione-targeted nanocarriers	Codelivery platforms for targeted killing by inducing chemosensitivity.	[121]
Transferrin-linked polymeric nanocarriers	DOX release in the intravacuolar compartments following endocytosis, favoring better targeting efficiency against leukemia	[122]
Polydopamine-layered zein nanocarriers	Increased cellular uptake, ROS production, and induction of apoptosis in the glioma cells, approaching efficient therapy against GBM.	[123]
Poly-L-lysine-based SEDDS	Proficient targeting with eradication of *Salmonella typhi* and 100% survival.	[124]
Vancomycin-loaded thiolated nanocarriers	Effective targeted ocular delivery system against *Staphylococcal blepharitis* with improved retention time, sustained drug release, and targeted anti-inflammatory action.	[125]
Arginine-based nanocarriers	Decreased bacterial burden and increased survival because of synchronized antibacterial, targeted, and ROS cellular response against *S. typhi*.	[126]

DOX: doxorubicin; ROS: reactive oxygen species; GBM: glioblastoma multiforme; SEDDS: self-emulsifying drug delivery system.

## Data Availability

Data sharing is not applicable to this article as no new data were created or analyzed in this study.
